# Clustering-Based Energy-Efficient Self-Healing Strategy for WSNs under Jamming Attacks

**DOI:** 10.3390/s23156894

**Published:** 2023-08-03

**Authors:** Nicolás López-Vilos, Claudio Valencia-Cordero, Richard Demo Souza, Samuel Montejo-Sánchez

**Affiliations:** 1 Synopsys Lisboa, 2740-267 Porto Salvo, Portugal; nicolasl@synopsys.com; 2 Department of Electrical Engineering, Universidad de Santiago de Chile, Santiago 9170124, Chile; claudio.valenciac@usach.cl; 3 Department of Electrical and Electronics Engineering, Federal University of Santa Catarina, Florianópolis 88040900, SC, Brazil; richard.demo@ufsc.br; 4 Programa Institucional de Fomento a la I+D+i, Universidad Tecnológica Metropolitana, Santiago 8940577, Chile

**Keywords:** clustering, energy efficiency, Internet of Things, jamming, power control, self-healing, security, wireless sensor networks

## Abstract

The Internet of Things (IoT) is a key technology to interconnect the real and digital worlds, enabling the development of smart cities and services. The timely collection of data is essential for IoT services. In scenarios such as agriculture, industry, transportation, public safety, and health, wireless sensor networks (WSNs) play a fundamental role in fulfilling this task. However, WSNs are commonly deployed in sensitive and remote environments, thus facing the challenge of jamming attacks. Therefore, these networks need to have the ability to detect such attacks and adopt countermeasures to guarantee connectivity and operation. In this work, we propose a novel clustering-based self-healing strategy to overcome jamming attacks, in which we denominate fairness cooperation with power allocation (FCPA). The proposed strategy, aware of the presence of the jammer, clusters the network and designates a cluster head that acts as a sink node to collect information from its cluster. Then, the most convenient routes to overcome the jamming are identified and the transmit power is adjusted to the minimum value required to guarantee the reliability of each link. Finally, through the weighted use of the relays, the lifetime of each subnetwork is extended. To show the impact of each capability of FCPA, we compare it with multiple benchmarks that only partially possess these capabilities. In the proposal evaluation, we consider a WSN composed of 64 static nodes distributed in a square area. Meanwhile, to assess the impact of the jamming attack, we consider seven different locations of the attacker. All experiments started with each node’s battery full and stopped after one of these batteries was depleted. In these scenarios, FCPA outperforms all other strategies by more than 50% of the information transmitted, due to the efficient use of relay power, through the weighted balance of cooperative routes. On average, FCPA permits 967,961 kb of information transmitted and 63% of residual energy, as energy efficiency, from all the analyzed scenarios. Additionally, the proposed clustering-based self-healing strategy adapts to the change of jammer location, outperforming the rest of the strategies in terms of information transmitted and energy efficiency in all evaluated scenarios.

## 1. Introduction

Wireless Sensor Networks (WSN) facilitate communication, control, and understanding with the surrounding world. Consequently, the WSNs are being deployed in several environments owing to their capabilities [1]. These characteristics, in combination with the possibility of the nodes being connected to the Internet, constitute the base for the Internet of Things (IoT) paradigm [2,3]. A WSN integrates numerous sensors, nodes, routers, and gateways to communicate data along the network. Moreover, any node in the network can access the Internet and be managed remotely. Also, such architecture allows those authorized to reach the network to access the data handled by the nodes [4]. Therefore, the integrity and reliability of the data are crucial to the objectives for which the WSN was created.

A WSN deals with numerous challenges to guarantee the reliability, energy efficiency, and correctness of the data in the network [5]. Due to the broadcast nature of WSN, attackers take advantage of this to weaken and compromise the network performance [6]. Specifically, the attack strategies that disturb the physical layer (PHY) are detrimental to the data. These types of attacks can be categorized as eavesdropping and jamming. Both attacks aim to take advantage of the data for the convenience of the attacker. Nevertheless, self-healing techniques have been created to overcome and relieve the impact of the attackers on the integrity of the data [7].

The self-healing techniques take advantage of the properties of WSN, such as rerouting, power allocation, and cooperation, to guarantee the reliability of the network and data. This technique is closely related to the clustering techniques. Both approaches adapt the network topology to ensure reliability, energy efficiency, and data integrity, among other objectives. However, the joint use of these techniques to overcome jamming attacks to guarantee the communications in the network under attackers’ scenarios is an unexplored topic. For this reason, this paper proposes a novel approach to overpower the presence of jamming attacks in several scenarios.

The novel approach uses the strengths of self-healing and clustering techniques to mitigate the intrinsic drawback of each one and guarantee the communications in the network under jamming attacks. Load balance, rerouting, cooperation, and power allocation are used. Then, we compare the energy efficiency and data correctness between several techniques in jamming scenarios. The exhaustive comparative analysis provides valuable insights.

The remainder of the paper is structured as follows. Section 2 provides a detailed and exhaustive review of the state of the art of works focused on clustering, jamming, and self-healing. Next, the contributions of our work are listed and presented to remark on the findings and contributions to the unexplored topic. Then, Section 3 provides a detailed description of the system model. Next, in Section 4, we discuss the applicability of different clustering strategies under jamming. Later, in Section 5, we describe our novel proposed algorithm to overcome the jamming attacks in the WSN context using different techniques. Then, in Section 6, experimental data is discussed. Consequently, Section 7 discusses, explains, and details the main remarks from the scenarios. Finally, Section 8 concludes the paper and considers some directions for future work.

## 2. Related Work

The use of clusters in combination with self-organization and automatic configuration algorithms can overcome diverse challenges in the reliability and security of wireless networks [8,9,10,11,12,13,14,15,16,17,18,19]. In this sense, the most used clustering protocols are LEACH [8] and its variants: HEED [9], PEGASIS [10], EECS [11], and TEEN [12]. By the use of hierarchical routing based on clustering, these protocols guarantee data communication with energy efficiency. The principal differences among the protocols are based on the selection of the CHs, the hops between the nodes, the construction of the routing path, and the cost function to select the nodes. In addition, the security problem is not considered in the creation of these clusterization protocols.

Some security-oriented LEACH variants have also been proposed, such as SLEACH [13], SecLEACH [14], Armor-LEACH [15], and MS-LEACH [16]. In [13], the authors propose the use of two symmetric keys for each node that are shared with the gateway to improve the authentication process. However, the authors of SecLEACH [14] state that SLEACH does not provide a complete and efficient solution for node-to-CH authentication problems. By the analysis of compromised links, the authors show that their proposed scheme improves security. However, energy efficiency is not completely met due to the generation of key pools and their distribution in the network. To improve the energy efficiency of SecLEACH, Armor-LEACH is proposed [15]. Using the SecLEACH algorithm and the Time-Controlled Clustering Algorithm (TCCA) as the basis, the security and energy efficiency of the network improves.

The MS-LEACH protocol [16] combines single-hop and multi-hop transmissions, which results in an improvement in the network lifetime with respect to LEACH. Nevertheless, jamming is not taken into account. The authors of SEC in [17] propose a combination of SPINS [18] and LEACH protocols to improve network security. With the addition of data authentication and data freshness, the gateway is capable of verifying the authenticity of the data and CHs. However, neither work considers the possibility of jamming attacks and nor take into account performance metrics such as information transmitted or energy efficiency. Finally, the Enhanced SLEACH protocol is proposed in [19]. With the use of pairwise keys among the cluster members and their respective CHs, it outperforms SLEACH in security, lifetime, and energy consumption aspects. However, as it happens with all the above approaches, it does not consider the presence of jamming attacks and their impact.

Moreover, in the last few years the use of clustering for energy-efficiency maximization has been extensively investigated [20,21,22,23,24,25,26,27,28,29,30,31,32]. The works in [20,21,22,23,24,25,26,27,28] focus on the residual energy (or remaining battery power), while they coincide in that the cluster head (CH) selection is critical to extending the network lifetime. Using Bee Colony Optimization [20], Fuzzy Logic [21,28], Butterfly Model [22], Hierarchical Clustering [23], Firefly Algorithm [24], Rider Optimization Algorithm (ROA) [25], Particle Swarm Optimization [26] and Fog Logic [27], these works improve the CH selection in different scenarios. On the other hand, some works focus on clustering and partition problems [29,30]. Using machine learning techniques and proposing novel clustering routing protocols, they aim at balancing the energy efficiency of the routing path. However, none of these works deal with security or jamming. The authentication issue is considered in the IoT context [31,32]. However, the problem of authentication in the context of the cluster with a focus on energy efficiency is only considered in [31]. In [33], jamming is considered in a specific clustering context. The work proposes a novel strategy that aims to detect and eliminate the jammer node present in the network to improve energy efficiency. Using the packet data rate (PDR), round trip time (RTT), packet loss ratio, and RSS, the hyperbolic spider monkey optimization (HSMO) algorithm detects and eliminates the jammer node. However, the work does not consider a specific jamming strategy or model the jammer node. Therefore, the PHY parameters or channel models are inexistent owing to the focus of the work. Additionally, this work assumes that the edge and sub-edge nodes use a different frequency channel to communicate, which is no minor requirement.

The cooperation scheme in the WSNs context is used primarily to improve energy efficiency [34,35]. Cooperation schemes are based on the use of multi-hop communications. The evolution of these schemes has demonstrated the benefits of the selection of relays, which required considering the resources available by the nodes. In [34], the authors proposed a cooperative scheme in which the relays activate the receiving circuitry based on switching probability. Then, the ON-OFF probabilities were adjusted to reduce power consumption and maximize the effective information transmitted. This proposal was improved in [35] by considering the use of multiple antennas in the sensor nodes, as well as power control. However, neither proposal considers how to keep the network running during a jamming attack.

On the other hand, the relationship that has been established today between WSNs and unmanned aerial vehicles (UAVs) is undeniable [36,37,38]. In [36], a comprehensive review is provided of the main applications of WSNs, UAVs, and monitoring technologies. The mobile sink-based solutions have triggered the investigation of UAVs as data mules. In [37], the authors addressed the problem of optimizing the UAV path through all the sensor nodes distributed over a large agricultural area to reduce its flight time and increase the nodes’ lifetime. In addition, the authors proposed an efficient algorithm for discovering and reconfiguring the activation time of the nodes. Meanwhile, in [38], an energy-efficient and fast data collection scheme was designed in UAV-aided WSNs for hilly areas with the help of a UAV as a data mule. The authors applied a modified tabu search algorithm to optimize the UAV position to collect data from a group of nodes and the traveling salesman problem to achieve fast data collection. We consider the crucial role of UAVs in smart outdoor applications such as agriculture, transportation, health, and public safety. Therefore, the parameters determined in this work consider the possibility of using UAVs to perform determined functions.

However, we wish to emphasize that the assistance of WSNs by UAVs does not guarantee the operation of the network against jamming attacks, as shown in [39,40]. In [39], the authors propose an anti-jamming scheme for collecting data in the presence of jamming attacks. A clustering approach is used to minimize the points that UAVs visit and guarantee that the message transmitted from the cluster heads reaches the UAV. The probabilistic channel model presented and the constraints imposed on the UAV and devices suggest that the jamming attack can be a constant type. The results show that the proposed approach surpasses the optimal solution, min k-means and genetic algorithm, and genetic algorithm without clustering. Nevertheless, the work does not consider the link communication between the CH and the clustered nodes. Therefore, clustering strategies such as cooperation or power allocation are not considered. Moreover, the energy efficiency of the information transmitted is not part of the study. On the other hand, in [40], a similar approach to improve the data collection and trajectory of the UAV on the network is employed. Jamming is considered, but from the defensive side as a countermeasure. Meanwhile, from the attacker’s side, only eavesdropping is used. Consequently, the jamming attacks in network communications are not part of the study.

### Contributions

Clustering techniques with self-healing capabilities have not been completely investigated in the literature. Moreover, previous works only analyze such techniques in scenarios free of attackers. In this article, unlike previous works, we analyze the performance of different strategies in WSNs under jamming attacks. In addition, we propose a novel strategy that combines clustering, power allocation, cooperation, and load-balancing techniques to ensure network self-healing. Using the metrics of residual energy, network lifetime, coverage, and amount of transmitted information, we analyze the potential of combining these techniques under jamming attacks. Thus, the main contributions of this paper are as follows:

- We analyze different clustering and self-healing techniques with power allocation and cooperation capabilities in scenarios with jamming attacks. For each scenario, we provide a detailed qualitative and quantitative analysis of the algorithms and techniques with their strengths and weaknesses.
- We propose a novel adaptive clustering-based self-healing algorithm that combines power allocation, cooperation, and load balancing. This novel algorithm is tested in the previously ignored presence of a jamming attack, ensuring efficient network operation in such conditions.
- We describe the advantages and disadvantages of each technique in jamming scenarios in terms of residual energy and transmitted information. The exhaustive analysis of routing paths, energy efficiency, and transmission powers insightful information on the behavior of the network against jamming attacks.

In Table 1, we highlight those related works that have mostly addressed the issues dealt with in this paper. Only the method proposed here addresses jamming with a multidimensional perspective, exploring clusters to efficiently control the transmit power, to self-heal and to maintain persistent operation despite the presence of jamming.

## 3. System Model

**Figure 1:**
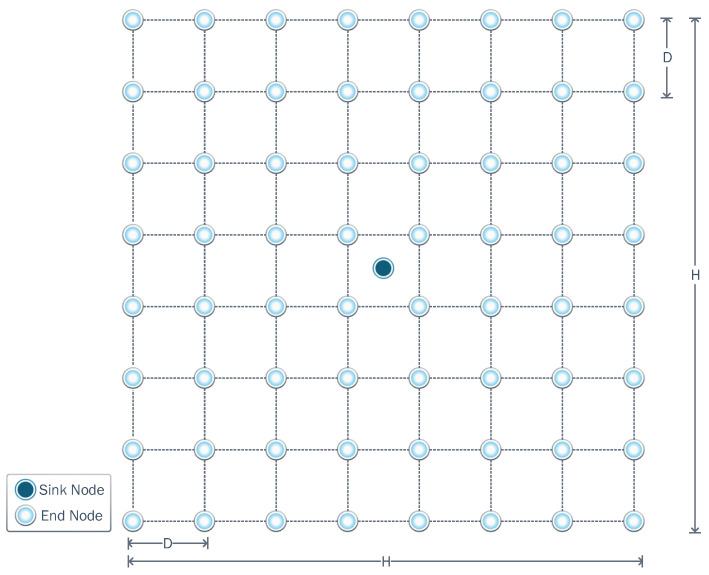
A network composed of *N* sensor nodes distributed in a square area with *H* edge-length and equal space *D* between nodes. To improve the readability of the nodes and positions on the network, we use a chessboard approach to simplify when we speak about a particular position along the work.

We assume *N* sensor nodes are distributed in a flat square area of H×H meters. The sensor nodes communicate with a gateway (or sink) node located in the center of the square area, as shown in Figure 1. The distance of pairwise nodes is expressed as *D*. The WSN nodes use time division multiple access (TDMA). Moreover, the nodes are static, and their positions are known to all nodes. This information allows each node to execute clustering algorithms in a distributed way and previously know the most convenient associations. When the system operates in cooperative mode, all the nodes can assume a relay role and use TDMA to transmit their data. Additionally, we assume that nodes can implement techniques that allow them to estimate the presence and location of a static jammer. Although this point is out of the scope of this work, note that nodes can implement energy detection techniques and share this information with neighbors, which would allow the execution of location techniques. For a better understanding of jammer localization in multi-hop wireless networks, the following survey [41] can be consulted. Moreover, there are recent jammer location techniques that are efficient even in the face of jammer mobility [42] and of multiple jammers [43].

The scenario considers a flat outdoor environment. We assume that no obstacles are in the line-of-sight (LOS) between the nodes. Moreover, we assume that the jammer’s location is determined by its decision, but we limit ourselves to considering the most representative potential locations to carry out a viable number of experiments.

It might be thought that the self-healing capability is not required for WSNs, considering that a UAV can fly over each sensor node and collect information from it, as described in [37]. However, from the analysis of [37], we can conclude that collecting information from all nodes in a network is an energetically expensive task, especially in WSNs with many nodes. In such scenarios, guaranteeing the freshness of the information would be an infeasible task for a single UAV. Therefore, if a network is attacked and its nodes are isolated from the sink, it is convenient for the network to be capable of self-healing, at least in interconnected groups, in such a way that the UAV would only have to collect information from the leader of each group.

### 3.1. Channel Model

The log-distance model is a suitable channel model for this flat scenario with small distances between nodes [44], in which the received power is calculated as

(1) $$P_{r}=\frac{kP_{t}}{d_{tr}^{\alpha }},$$

where $P_{t}$ is the transmit power, $d_{tr}$ is the distance between transmitter and receiver, α is the path-loss exponent, and *k* accounts for other factors such as the wavelength, height, and antenna gains [44,45]. Consequently, the signal-to-interference-plus-noise ratio (*SINR*) perceived by a receiver in the presence of a jammer when the *i*th node transmits is

(2) $$SINR_{i}=\frac{kP_{i}d_{ir}^{-\alpha }}{kP_{j}d_{jr}^{-\alpha }+n_{0}W},$$

where $P_{i}$ and $P_{j}$ are the transmit powers of the *i*th node and jammer, respectively; $d_{ir}$ and $d_{jr}$ are the distances from the *i*th node and the jammer to the receiver, respectively; $n_{0}$ is the noise power spectral density, and *W* is the channel bandwidth. Therefore, if the transmit power and location of the jammer are estimated, it is then possible to estimate the minimum transmit power ( $P_{i}^{*}$ ) required by the *i*th node to satisfy the *SINR* threshold ( $\gamma_{0}$ ) at the receiver, according to a maximum transmit power ( $P_{\max}$ ) constraint,

(3) $$P_{i}^{*}=\frac{\gamma_{0}\left(kP_{j}d_{jr}^{-\alpha }+n_{0}W\right)}{kd_{ir}^{-\alpha }},\;s.t.\;P_{i}^{*}\leq P_{\max}.$$

### 3.2. Jamming Attacks

Every communication system that works with data is exposed to security threats. Non-authorized users of the communication system may want to access, manipulate or destroy the data. In consequence, the communication system needs the timely identification of each attack class to neutralize them.

The WSNs may be exposed to two possible main attack classes: eavesdropping and jamming. In eavesdropping attacks, the spy user aims to passively listen to the information. This type of attack is normally combated by encrypting the information. On the other hand, jammers generate radio frequency signals in the same operation band and the main objective is to interrupt the system operation. The jammer can assume different operating strategies:

- Constant jammer: The radio signal from the jammer is emitted constantly in the communications channel. Consequently, the jamming signal and the legitimate signal collide almost all the time, which provokes the discard of the packets on the receiver side. However, this strategy demands excessive energy to perform the emission of the radio signal and it is easy to detect.
- Random jammer: The radio signal is randomly generated, decreasing energy consumption and making it difficult to predict the attack. Therefore, the probability of a collision is less than in the constant strategy but with higher stealth.
- Reactive jammer: This strategy takes advantage of the ability to hear the communication channel. The jammer device records the different sniffed data transmitted in the channel. Next, the attacker decides to emit the radio signal to target specific data packets. This capability improves energy consumption and stealthiness of the jammer.

A WSN may apply different strategies to defend against attackers. These strategies depend on the successful detection of attacks and the timely identification of the attack class to execute the defense mechanisms. Therefore, it is crucial to successfully identify whether an anomalous situation is due to an attack or other system factors. Several studies have shown that the use of metrics of received power, energy consumption, packet data rate (PDR), and bit error rate (BER) permit the detection of attackers in the majority of cases [46,47,48].

### 3.3. Energy Consumption Model

We consider the network lifetime as the operation time from when the batteries of the devices ( $B_{i}$ ) are full until the battery of any of the nodes is completely depleted. Since our research focuses on countering jamming attacks, for simplicity we assume that at the beginning of the attack, all devices had the same energy charge, equal to the total battery capacity, i.e., $B_{i}=B_{C}\;\forall \;i\in \left\{1,2,\cdots ,N\right\}$ .

IoT nodes consume energy in information acquisition and processing, reception and transmission of information, as well as other scheduling and synchronization functions [34,35,49]. However, we abstract in this research from all functions not related to communication, to compare the impact of different transmission control and/or cooperative communication strategies. The consumed energy for each process is calculated considering the meantime of the process and the components involved [34,35].

The energy consumed during transmission ( $e_{t_{i}}$ ) by node *i* can be estimated by considering its transmit power $P_{i}$ , the power amplifier efficiency η , the transmission circuit operating power $P_{ct}$ , and the transmission time, which is determined by the length of the message *L* and the transmission rate *R*,

(4) $$e_{t_{i}}=\frac{(P_{i}/\eta +P_{ct})L}{R}.$$

Similarly, for the reception, we calculate the energy consumption as

(5) $$e_{r_{i}}=\frac{P_{cr}L}{R},$$

where $P_{cr}$ is the energy consumed by the circuitry in the reception process. Therefore, the energy consumption of node *i* after transmitting $t_{i}$ messages is

(6) $$E_{T_{i}}=\sum_{j=1}^{t_{i}}e_{t_{i}}=t_{i}e_{t_{i}}.$$

The implementation of the self-healing strategies proposed in the following section implies that some nodes, designated as cluster heads, perform the function of information sinks, so their energy consumption depend on the number $r_{i}$ of messages received

(7) $$E_{R_{i}}=\sum_{j=1}^{r_{i}}e_{r_{i}}=r_{i}e_{r_{i}}.$$

Moreover, some of the proposed strategies are based on cooperative communication between nodes, so the energy consumption associated with the cooperation of these nodes depends on the number $c_{i}$ of messages in which they act as relays, receiving and re-transmitting the information,

(8) $$E_{C_{i}}=\sum_{j=1}^{c_{i}}\left(e_{r_{i}}+e_{t_{i}}\right)=c_{i}\left(e_{r_{i}}+e_{t_{i}}\right).$$

Therefore, the residual energy of node *i* depends on the self-healing strategy used, the role that the *i*-th node plays in its cluster, as well as the number of messages it receives (if it is a sink) or the number of messages it sends and those in which it cooperates (if it is a relay too). However, it should be noted that when transmit power control techniques are also used, the cost associated with forwarding the information will also depend on the relative distance of the node to which the information will be sent. Therefore, in such cases, if the relays of the next level change, the energy cost of sending the information to them changes as well.

## 4. Baseline Clustering Strategies under Jamming

In the face of a jamming attack, one of the first countermeasures must be to isolate the jammer. However, the location of the jammer is established by the attacker, and the most damaging position for the network is usually chosen, e.g., the sink node neighborhood. Therefore, it is often necessary to establish new sink nodes and clustering is used for this purpose.

Once the jammer is detected, its location and transmission power are estimated (which is considered resolved and out of the scope of this paper). Then, the cluster formation and selection of CHs is carried out. Using the K-medoids technique [50], we generate the clusters assigning an optimum number of centroids or CHs. For that sake we use as a metric the interference-plus-noise (IPN) that would be perceived from the location of each cluster node. The CH position must meet the following requirements to be chosen as valid:

- Reduce the attacker’s impact on the majority of the nodes in the cluster.
- Minimize the number of isolated nodes.
- Provide the highest number of associations.

The first two criteria can be incorporated into the clustering when considering the Chebyshev distances [50] and the third one is used to select each CH. The clustering process itself leads to a trade-off between two options: many small clusters whose internal communication is not affected by the jammer; and a larger number of clusters that allows data collection from emerging sink nodes, e.g., through a UAV.

The clustering strategies that we propose take into account both purposes, which does not guarantee that all nodes of the same cluster can communicate directly and successfully with the selected CH using the same transmit power as when there is no jammer. To guarantee effective communication in such cases, next, we discuss baseline strategies exploiting transmit power control, cooperative communication, and a combination of both.

### 4.1. Power Domain

The use of optimum transmit power improves energy efficiency and extends the network lifetime. Therefore, the comparison between strategies that use fixed power (FP) in all nodes with strategies that use power allocation (PA) conveniently is the first starting point to establish benchmarks in our research.

However, in practice the utilized hardware and/or legislation may impose upper and lower limits of potential transmit powers. Therefore, in some cases, the necessary power to transmit and ensure the total reception of the messages cannot be ensured. Consequently, some messages will be lost by the effect of the disruption signal generated by the jammer. Based on [47,51], exists an *SINR* threshold that permits decoding the majority of packets correctly. Therefore, meeting this requirement according to the established maximum transmit power constraint determines the nodes that can communicate directly with their CH in each cluster.

#### 4.1.1. Fixed Power (FP)

In this strategy, we assume that clustered nodes use fixed transmit power to reach the CH. Therefore, the transmit power is chosen to ensure that the nodes communicate with the CH in a single hop. Setting the transmit power fixed to the required upper limit guarantees intra-cluster communication, but implies unnecessary power consumption for many of the established links.

To illustrate the energy cost of this strategy, we establish a fixed transmit power for all nodes that guarantee communication. Therefore, the communication link between the node most affected by the jammer and the corresponding CH will be the constraint in the transmit power. Consequently, the fixed transmit power is acquired according to (3).

#### 4.1.2. Power Allocation (PA)

Instead, this strategy assumes that the nodes can individually set their transmit power to reach their respective CH. Therefore, each node calculates the optimum transmit power that ensures the correct reception of the message and the CH, according to (3). Consequently, the nodes closer to the CH can use lower transmit powers and reduce their energy consumption. In contrast, the nodes far from the respective CHs demand higher transmit powers, and eventually, they drain their batteries quicker.

### 4.2. Cooperation Domain

In a WSN, the nodes may assume different roles in the network. By varying the role of the nodes and making new links between them, several new communication paths can be created.

#### 4.2.1. Cooperation (CP)

In the cooperation case, the nodes can retransmit their data using relay nodes until reaching the CH. However, they use a fixed power. The nodes evaluate the best cooperation route using the Minimum Receiver Sensitivity (MRS) metric to build the retransmission chain. Several nodes can cooperate in this scheme, but the selection between the different routes is based on the *SINR* value of the weakest link of each route. Once the link with the lowest *SINR* in each route has been identified, the *SINR* values of these worst links in each route are compared and the route corresponding to the one with the highest *SINR* is chosen, i.e., the route with the best worst link. Since in this scheme it is assumed that all nodes use the same transmit power, the smaller (greater) the Euclidean distance between nodes, the greater (smaller) the *SINR*, as long as the distances from the jammer to the potential relays are similar.

Summarizing, for the cooperation scheme, we have the following considerations and assumptions

- All the nodes can assume the role of a relay node in the routing chain.
- The Euclidean distance is used to find the optimum route considering the distance and hops between the isolated nodes and the CHs.
- If two cooperating nodes have the same Euclidean distance, the first node found by the algorithm is chosen.

In this strategy, the nodes that by direct connection with the CH meet the *SINR* threshold (i.e., those that can directly transmit their messages) are considered associated. Then, each isolated node evaluates if the closest associated neighbor serves as a relay, according to the *SINR* of that link. As new nodes become associated, those isolated ones evaluate new associations that imply more hops. This action is repeated until all nodes can associate or at least all nodes that can associate according to the preset transmit power.

#### 4.2.2. Power Allocation and Cooperation (PAC)

The fourth strategy uses both techniques, cooperation and power allocation, to route the data. Therefore, the nodes that participate in the cooperation optimize the transmission power to reach the next relay node. Consequently, the nodes involved in the routing chain can reduce their energy consumption and increase their lifetime.

## 5. Fairness Cooperation with Power Allocation (FCPA)

**Figure 2:**
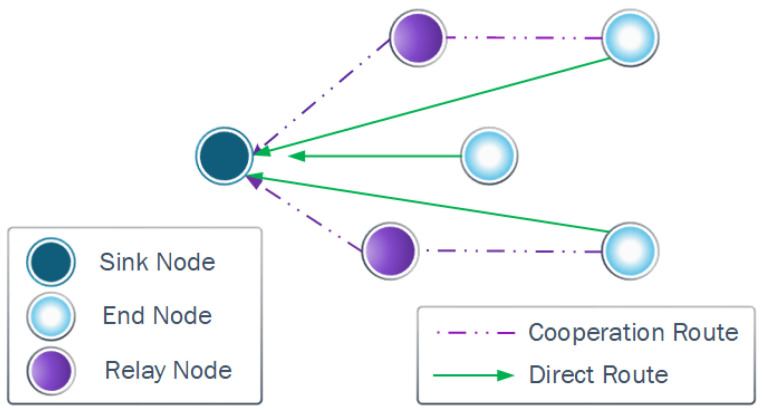
Different routes that the nodes can use to reach the sink node in the network using direct routes or cooperation routes.

Note that some relay nodes in a WSN may significantly reduce their lifetime to promote cooperation. Therefore, we focus on a strategy that allows load balancing between potential relays and potential paths, as shown in Figure 2.

The previous strategies used to overcome the jamming problem provide different approaches to ensure that the isolated nodes reach the sink node or the CH. However, the constraints imposed do not consider energy efficiency, coverage, and the fulfillment of their objectives. Consequently, an algorithm that improves the rerouting path construction and the load balance in energy terms will also contribute to these objectives. To this end, we analyze the weakness of the presented protocols to propose a novel protocol that solves these problems. We call this novel protocol Fairness Cooperation with Power Allocation (FCPA).

Thus, next, we propose a novel protocol that permits the relay nodes to cooperate and maximize their energy efficiency in the routing process.

### FCPA Protocol

The Fairness Cooperation with Power Allocation (FCPA) protocol assigns weights to the cooperating nodes to distribute the load. The allocation of weights considers the energy cost of reception and retransmission associated with cooperation.

The lifetime of the network is maximized using the following modifications and criteria:

- Modifications in the network, such as the appearance of a jammer, its mobility or transmit power change, demand that each isolated node chooses a CH as its sink node using the Euclidean distance that separates it from the CH and the CH from the jammer. Likewise, it chooses the closest neighbors associated with the cluster of the chosen CH.
- Since the association of nodes to a CH is subject to minimizing the energy cost associated with communication, when the location of the jammer changes the nodes that remain isolated may conveniently associate with a different CH. Consequently, the number of nodes associated with each cluster may change.
- The selection of each relay node is conditioned by the number of hops between the optimum route chosen in the cooperation algorithm. If any route exceeds the preset number of hops limit $N_{hops}$ , then it is discarded. This maximum allowed number of hops is preset by the network manager.
- The weight associated with the relay selection is inversely proportional to the energy cost of the cooperation and is determined by the number of alternative valid routes to support the same communication.

**Figure 3:**
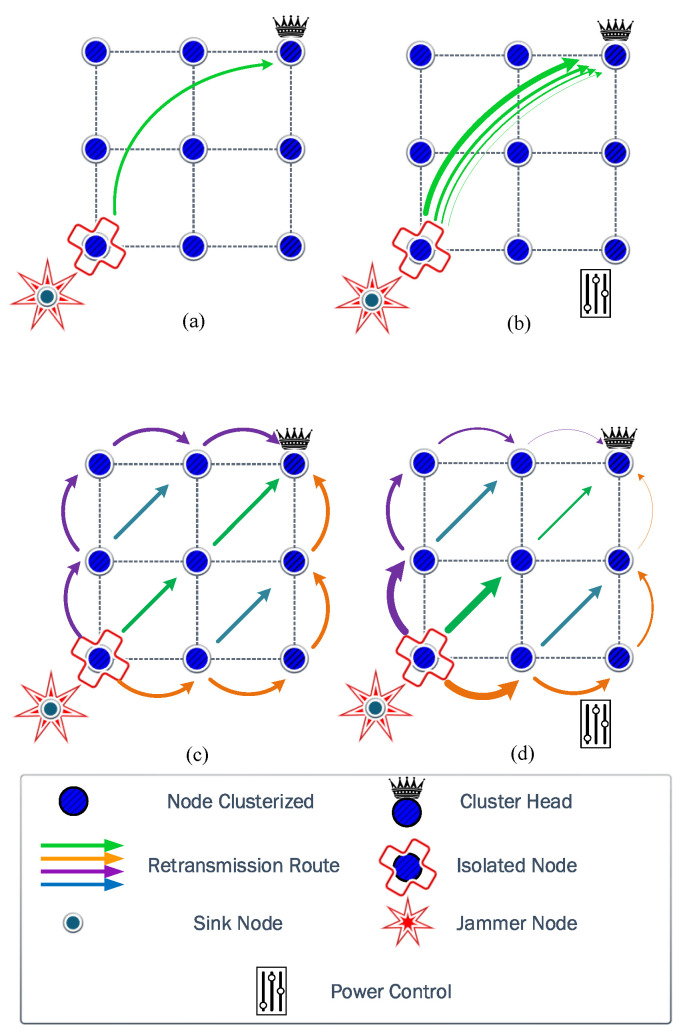
The arrows show the different routes from the isolated node to the cluster head. A wider arrow represents a higher transmit power than a thinner arrow. In (**a**), the direct communication with fixed transmit power is executed by FP, while in (**b**), the power control is used by PA. The inclusion of cooperation occurs for (**c**) by CP, where different routes are considered with the same transmit power. In (**d**), the transmit power control and cooperation algorithms are integrated.

The weights used to distribute the load between the cooperating nodes are estimated as

(9) $$w_{i,j}=\frac{1/e_{cop_{i,j}}}{\sum_{k=1}^{K}\frac{1}{e_{cop_{i,k}}}},$$

where $e_{cop_{i,j}}$ is the cooperation energy between the *i*th node that needs cooperation and the *j*th node (relay) that provides the cooperation. Then, $e_{cop_{i,k}}$ is the energy of the *k*th node that can provide cooperation to the *i*th node in the routing chain, where *K* is the number of alternative valid routes of the *i*th node. Finally, this approach generates a tree-based clustering given the distribution of the load as shown in Figure 3.

## 6. Simulation Results

In this section, we present comparative results of FCPA against the baseline power control and cooperation strategies discussed in Section 4. We evaluate the performance of the self-healing protocols in a WSN under constant jamming attack in terms of energy consumption, the lifetime of the network, coverage, and PDR. To this end, we simulate the protocols in an outdoor and flat coverage area using MatLab R2021b software. Table 2 shows the system parameters used in the validation and evaluation phase. The simulation runs until the first node exhausts its battery, then the data referring to residual energy and collected information are processed.

**Figure 4:**
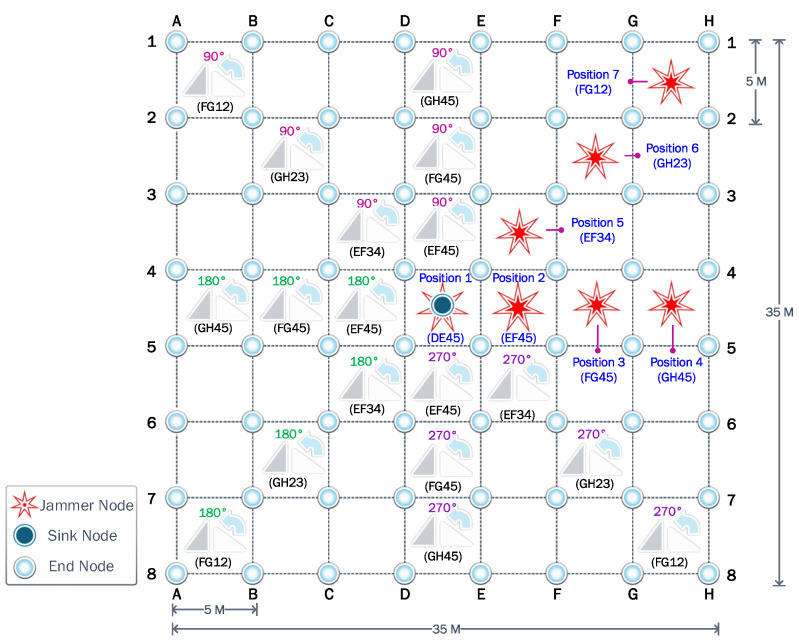
Evaluation scenarios: Wireless sensor network with 64 static nodes, and the seven jammer locations considered in the research.

We consider a WSN with 64 nodes uniformly spread, as shown in Figure 4. In the absence of a jammer, it is logical to assume that the sink node is located at the center of the WSN region. Therefore, the most harmful location for a jammer would be that same position, thus annulling the operation of the sink node. That is why first we consider the jammer at the center, the so-called Position 1. Then, to evaluate the impact of the jammer location, always assuming the cancellation of the initial sink node, we consider another six relevant positions according to the symmetry of the proposed scenario. Consequently, the performance of the different strategies is evaluated for each scenario according to the seven potential jammer locations. In all cases, the jammer transmit power is considered fixed and equal to 14 dBm.

In the results below, we show the clusters and network structures associated with each self-healing strategy in response to the jamming attack in each scenario according to the self-healing strategies. In addition, we provide data referring to the amount of information transmitted by the WSN and the residual energy of each node.

### 6.1. First Scenario

**Figure 5:**
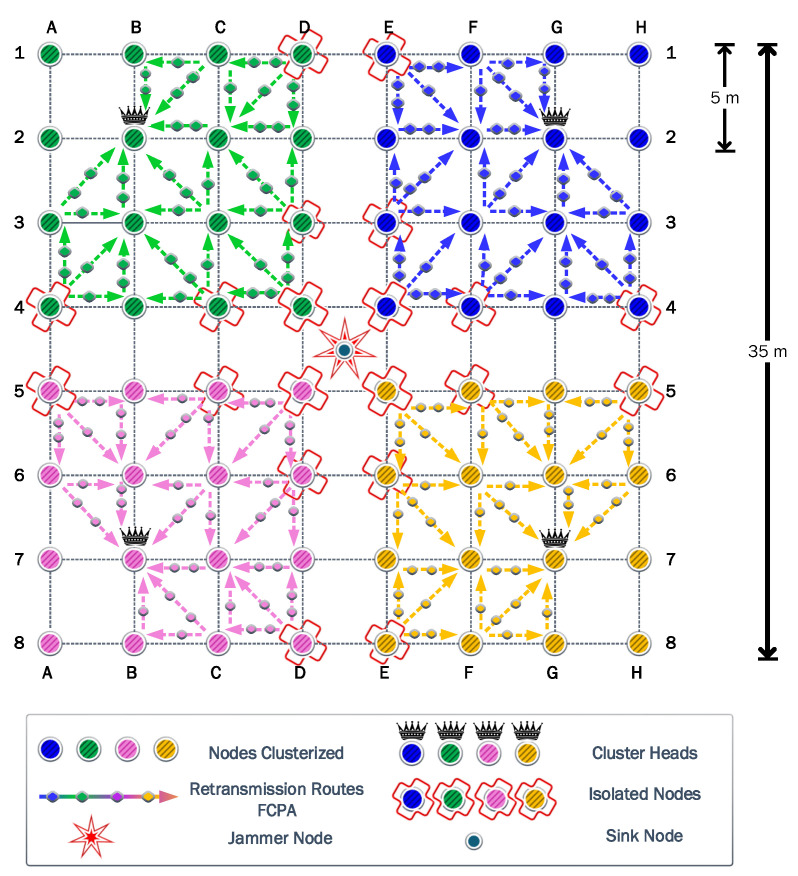
First scenario: the clustered network is represented with the CHs of each cluster, the jammer at Position 1, and the assisted communications when using cooperation-based strategies.

The first experiment uses a static jammer deployed in the middle of the network, in the same position as the sink node in DE45. Then, we assess the clustering and self-healing algorithms to evaluate the effectiveness of these strategies. Firstly in this scenario, it was determined that four is the optimal number of clusters. These four associated clusters are illustrated with different colors in Figure 5. Next, the CH of each cluster was selected considering the Chebyshev distance. Then, each node associates with the CH that guarantees the highest *SINR*.

Figure 5 shows that when the jammer is located in the center of the network, the nodes are clustered into four quadrants of 16 nodes each, while their respective CHs are the nodes located in B2, G2, B7, and G7. It also shows that some nodes are isolated since they cannot communicate directly with their respective CHs, due to transmit power limitations. The symmetry of this first scenario allows a more detailed analysis to be discussed based on a single cluster.

To keep the clearness and simplicity of the FCPA algorithm analysis, Figure 5, as well as the figures corresponding to the other scenarios, show the rerouting paths of the isolated nodes to the corresponding CH. But, the routing path of nodes associated with the corresponding CH is omitted.

**Figure 6:**
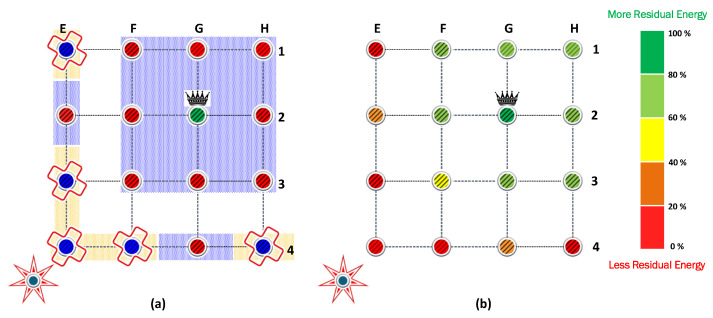
Residual Energy for (**a**) the FP algorithm with 14 dBm of fixed transmit power and (**b**) the PA algorithm with 17 dBm of maximum transmit power.

Figure 6a shows the result of the FP algorithm from Section 4. Note that when the transmit power of the nodes and the jammer is the same, some nodes cannot communicate directly with the CH. The blue background nodes are associated nodes, while the nodes with the orange background are isolated nodes, which represent a third of the nodes of each cluster. Additionally, the residual energy from each battery is denoted by the gradient color bar. Operating all nodes with 14 dBm of transmit power is very energy inefficient. When all associated nodes have exhausted their batteries, the battery of the sink node remains with a high energy load. On the other hand, when the power control is activated, we verify that transmitting with 17 dBm allows all the nodes to communicate directly with the CH, even the E4 node, which is the most distant from the CH in the analyzed cluster. To ensure that the comparison between all algorithms is fair in terms of providing coverage to all nodes on each cluster, the non-cooperative FP and PA strategies will use 17 dBm as the maximum transmit power.

Figure 6b shows the result of the PA algorithm from Section 4. The transmit power allocation significantly favors the nodes closest to the CH, which can operate with low transmit power, so they remain with residual energy. However, it does not obtain benefits over FP in terms of network lifetime since this is determined by the node that operates with the highest transmission power (i.e., E4). Therefore, the network lifetime when FP and PA are used is the same, allowing only the collection of 804,528 bits.

**Figure 7:**
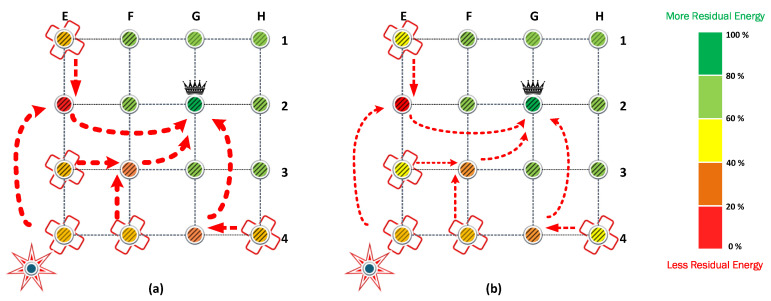
Residual Energy for the cooperative strategies, (**a**) the CP algorithm with fixed transmit power and (**b**) the PAC algorithm, where the power allocation is possible. The width of the arrows indicates when the power is fixed (**a**) and when it can be reduced to the value required by the link (**b**).

In strategies based on cooperation, the maximum transmit power was kept at 14 dBm to allow any node to be assisted by another node, even when it is not adjacent. Figure 7a shows the result of the CP algorithm from Section 4. CP provides coverage to all nodes even when the maximum transmit power preset is half of the preset in FP and PA. Now, the E4 node is assisted by the E2 node and does not need to reach the CH directly. When the CP algorithm is used, the first nodes that exhaust their batteries are E2, F3, and G4, which attend to the communications of the nodes isolated in Figure 6a.

The results show that the nodes that provide cooperation deploy their batteries first. Specifically, the nodes (E2, F3, G4) cooperate with the isolated nodes to retransmit their messages. Therefore, these nodes are under heavy load and are the first that exhaust their batteries. Next, the isolated nodes waste more energy than the CH because they require more power to transmit their message. Because of this, isolated nodes are the second nodes that exhaust their batteries, followed by CH. Finally, the nodes that reach CH in one hop are the last nodes that survive in positions (F1, G1, H1, H2, H3).

The scenario that combines cooperation and power allocation has similar behavior. The cooperation algorithm finds the same routes to retransmit the messages. However, in each link between the rerouting chain, the power allocation reduces the energy consumption in the transmission process. Therefore, the cooperating nodes are under a heavy load in contrast with the rest of the nodes in the cluster. Consequently, they can retransmit more information but exhaust their batteries first.

**Figure 8:**
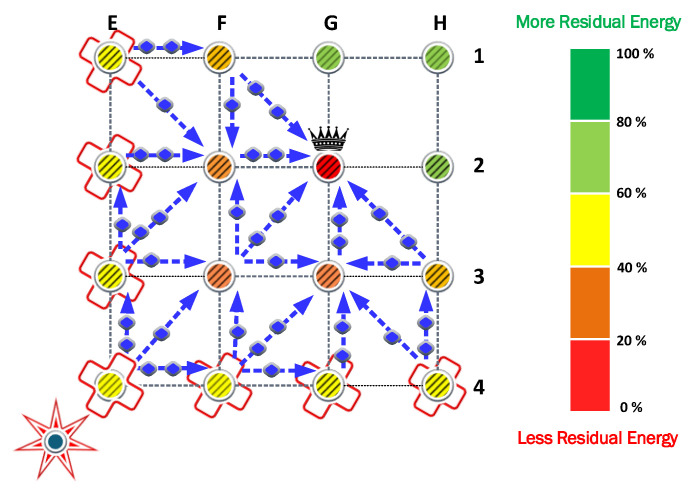
The FCPA strategy increases energy efficiency through combined power allocation and cooperation strategies. Consequently, the CH of each cluster is the first node that exhausts its batteries. We use arrows with markers to show the preference routes by the algorithm. Arrows with more markers indicate more used routes.

The FCPA algorithm takes advantage of the weakness of the previous protocols. Therefore, it provides relief to the nodes under heavy loads. To this end, the algorithm distributes the retransmission of messages to different routes. As a result, the number of nodes that cooperates increases, reducing the load for the nodes determined by the cooperation algorithm. In Figure 8, the routes and the relative load are represented by arrows and markers.

The FCPA algorithm significantly improves energy efficiency in the network. As presented in Figure 8, isolated transmitters and cooperating nodes distribute their loads equally. Consequently, the CH node is the first to exhaust its battery. This phenomenon is exclusive to this strategy and shows the robustness of the solution. Then, the cooperating nodes and isolated nodes keep relative residual energy, respectively.

**Figure 9:**
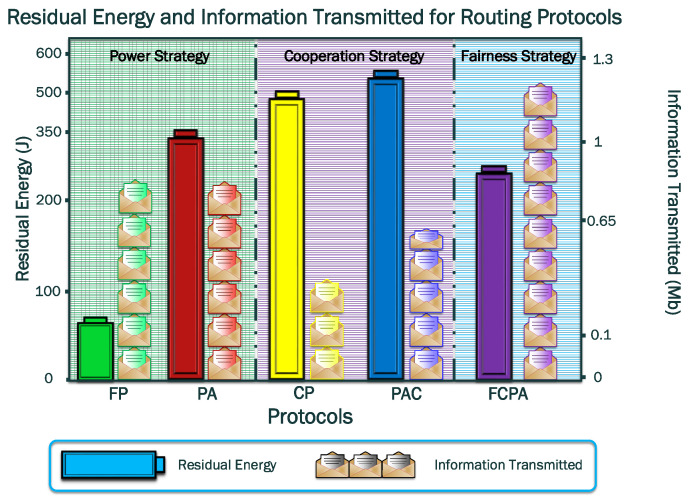
Evaluation of the different strategies according to the information transmitted (kb) and the residual energy (J).

In Figure 9, we present the results of the residual energy and information transmitted for the FP, PA, CP, PAC, and the proposed FCPA protocol. The results show that the tradeoff between the residual energy and the information transmitted in the FP is poor. The PA and CP protocols have a better performance than FP with 18.60 and 22.40 times more energy, respectively. However, the CP protocol has less information transmitted with 118,192 kb transmitted since, when operating all nodes with the same transmit power, the relay nodes deplete their batteries first. On the other hand, the PA strategy achieves the same amount of information transmitted as the FP, since the most distant nodes use the maximum transmit power. Therefore, the PA and CP protocols overpass the performance of the FP protocol in residual energy terms. Then, the combination of these two protocols named PAC improves the residual energy left in the network by 7% from the CP. Additionally, the amount of information transmitted increases to 139,158 kb from CP.

The amount of information transmitted is the same for the FP and PA protocols. This is explained because both protocols use the maximum power to reach the CH in one hop since the node closest to the jammer cannot reduce its transmit power. Therefore, in both strategies, this node will exhaust its battery first.

For the cooperation strategies, the amount of information transmitted and residual energy is slightly better for the PAC protocol than CP. In these strategies, the nodes selected as relays are under more demand. Therefore, the relay node that cooperates with a larger number of nodes will exhaust its battery faster than a node that does not cooperate. Consequently, the relay nodes in the first stage of the re-routing path, which provide the connectivity to the isolated nodes, present a higher load. Finally, our proposed FCPA strategy outperforms the performance of the other analyzed strategies in terms of residual energy and the amount of information transmitted. Its residual energy is less than other strategies, but the information transmitted is larger.

### 6.2. Second Scenario

**Figure 10:**
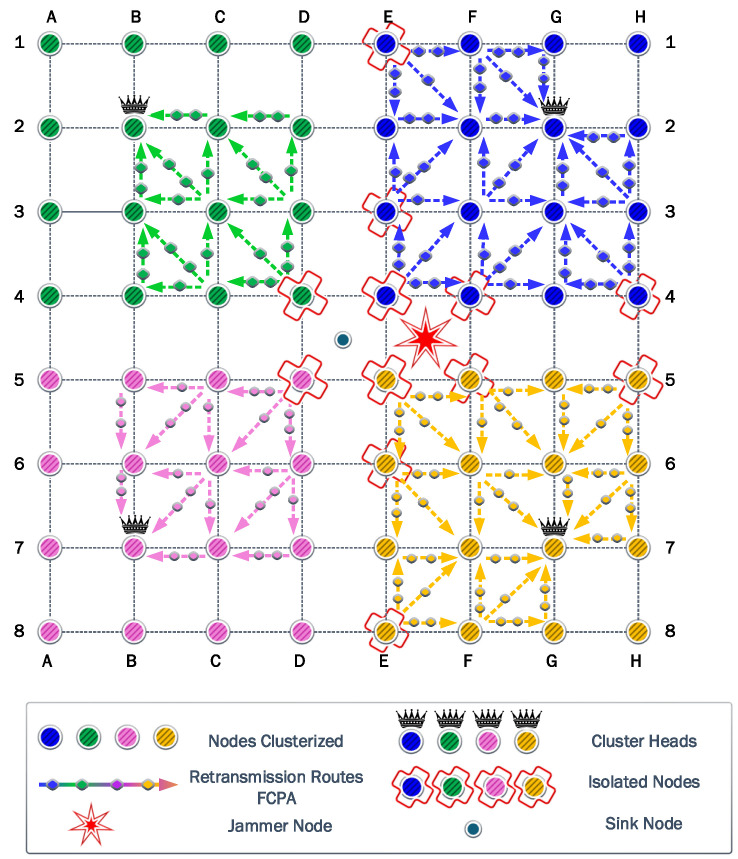
Second scenario: where the network adjusts to the presence of the jammer in the new position. Therefore, not all clusters present the same structure. The number of isolated nodes is higher in the blue and yellow clusters.

In this scenario, the jammer node is deployed in the second position to analyze the self-healing and clustering protocols. Figure 10 shows how the network reacts to the jamming attack. The clusters in green and pink colors adapt to the jammer keeping the same shape. However, the blue and yellow clusters reshape to overcome a strong jammer presence. Consequently, the coverage results are 93.30% for green and pink clusters and 66.67% for blue and yellow clusters.

The first steps of the algorithm try to reach the CH in one hop using the maximum power of transmission. Figure 10 represents the jammer effect on each cluster. The green and pink clusters have only one isolated node. On the other hand, the blue and yellow clusters have five isolated nodes in different positions. Then, the algorithm calculates the optimum transmission power to ensure all nodes reach the CH. The result of this calculation is a transmission power of 17.88 dBm.

The information transmitted for FP and PA strategies is the same, owing to the use of the same transmission power. The amount of information transmitted reaches 687,024 kb. Similar to the previous jammer position, the transmitter nodes deploy their batteries faster than the CH owing to the difference between the reception and transmission power. However, the PA strategy notably increases the residual energy by 423.77 J.

The cooperation strategy generates the same paths detected from the previous jammer position. The nodes that provide cooperation have less load owing to the decrease in the isolated nodes in green and pink clusters. However, relay nodes in blue and yellow clusters present the same load as the previous scenario. Consequently, the amount of information transmitted is the same for the CP strategy. Then, the PAC strategy marginally increases the amount of information transmitted to 120,720 kb. Additionally, the residual energy only differs by 26.95 J. Finally, FCPA surpasses all the protocols in the amount of information transmitted with 924,288 kb, while the energy residual is 396.97 J.

### 6.3. Third Scenario

In the third position of the jammer in the quadrant FG45, for the first time, the cluster most affected by the jamming effect chooses a new CH for each one. Blue and yellow clusters move their CH from G2 to F2 and G7 to F7, respectively. On the other hand, green and pink clusters preserve the previous CH.

**Figure 11:**
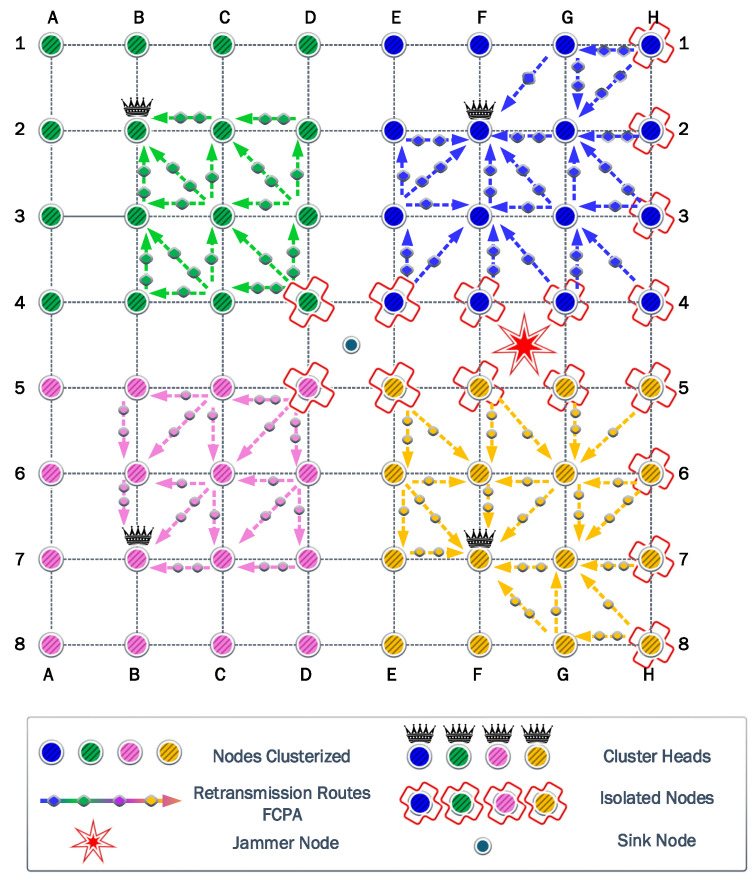
Third scenario: where the network adjusts to the presence of the jammer in the new position by the variation of the CH in blue and yellow clusters, increasing the number of isolated nodes for these clusters.

Additionally, isolated nodes in blue and yellow clusters increase to approximately 44% . Therefore, the cooperation and FCPA algorithms choose new routes and relays to achieve isolated nodes. Figure 11 shows the new relays and paths. For green and pink clusters, we observe that isolated nodes decrease to only one for each cluster. Consequently, the jammer significantly damages blue and yellow clusters.

The algorithm found a transmit power of 18.50 dBm to surpass the jamming effect based on the most affected nodes corresponding to H4 and H5. Therefore, the FP and PA strategies transmit 602,422 kb, while the residual energy is 11.76 J and 470.13 J for FP and PA strategies, respectively.

For the cooperation strategy, the nodes E3, F1, G2, G3, and H3 are defined as relays for isolated nodes. However, the relays E3 and G3 are the first nodes to exhaust their batteries completely. These nodes have a higher number of associations than the other relay nodes. Consequently, they spend more energy on each transmission and reception process. As a result, the residual energy is almost the same for CP and PAC strategies with 533.49 J and 536.93 J, respectively. For the information transmitted, we acquire 118,192 kb and 192,278 kb for CP and PAC, respectively. Then, the FCPA algorithm provides the best scenario again, the amount of information transmitted reaches 914,288 kb. The residual energy is 415.50 J. Therefore, the FCPA algorithm provides the best tradeoff between these metrics.

### 6.4. Fourth Scenario

**Figure 12:**
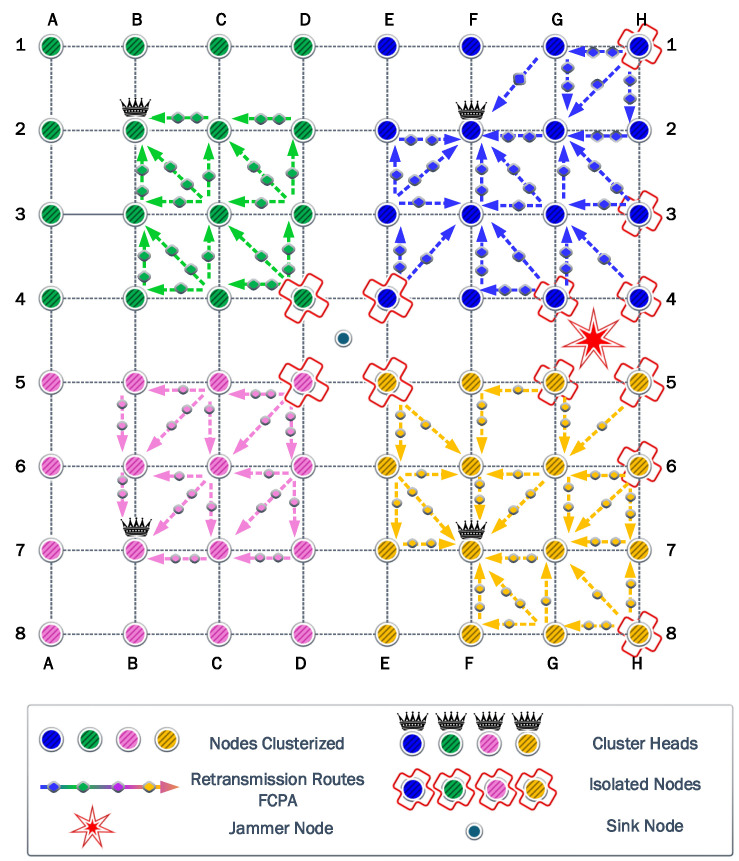
Fourth scenario: where the network chooses the same previous CH to provide communication on the clusters. However, the number of isolated nodes decreased significantly owing to the new position of the jammer.

Figure 12 shows that the CHs are preserved for the green and pink clusters when the jammer position is in the quadrant GH45. Again, the blue and yellow clusters are the most affected by the jammer. Additionally, the number of isolated nodes and their locations are the same. The nodes H4 and H5 are the nodes that suffer a higher impact from the jammer. However, the required transmit power to overcome the jamming is 17.90 dBm.

For the FP and PA strategies, the information transmitted increases to 687,024 kb, while the residual energy is 7.79 J and 462.53 J, respectively. As explained earlier, the cooperation strategies of CP and PAC have a poor performance in the information transmitted in the network, with results below 200,000 kb. However, in this jammer scenario, both strategies surpass this value and reach 245,868 kb and 387,763 kb for CP and PAC strategies, respectively. On the other hand, the residual energy is 465.77 J and 480.54 J, respectively. As in the previous scenarios, the difference is marginal for the residual energy. The FCPA strategy results show a notable performance, with the information transmitted being 914,288 kb and residual energy of 462.53 J.

### 6.5. Fifth Scenario

**Figure 13:**
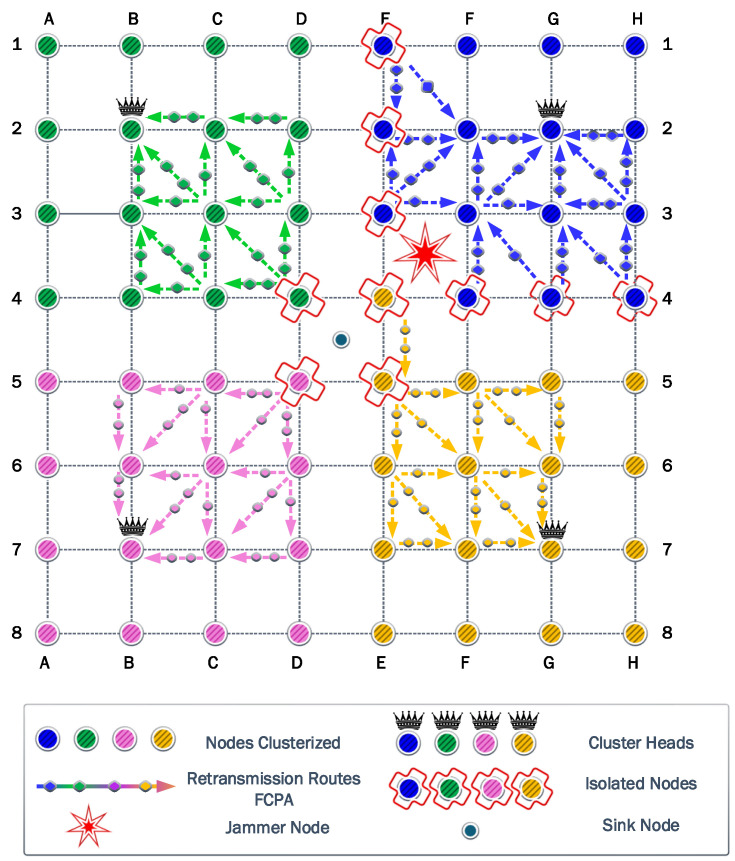
Fifth scenario: where the blue cluster is notably the most affected, with multiple isolated nodes.

The jammer in the quadrant EF34 provokes 38% of the isolated nodes in the blue cluster. Now, node E4 changes its cluster and CH association to the yellow cluster. This can be appreciated in Figure 13.

The clustering determines a power transmission of 19.05 dBm to surpass the jammer effect. This value is based on node E4, which requires this transmit power to reach the CH on position G7. Consequently, the amount of information transmitted for FP and PA strategies is 534,835 kb for FP and PA strategies. The residual energy is 14.92 J and 487.71 J, respectively.

The cooperation strategies choose nodes E2, F1, F2, G3, G4, and H3 as eligible relays. However, only relays F1 and H3 exhaust their batteries. Similar to the previous scenarios, these relays have more associations. Consequently, the amount of information transmitted reaches 245,869 kb and 300,262 kb for CP and PAC strategies, respectively. These results are similar to the previous scenario and demonstrate the impact of cooperation in this scenario. The residual energy acquired is 72.98% J for CP and 79.52% J for PAC. For the proposed strategy, FCPA, the information transmitted is 914,288 kb, while the residual energy is 72.27% . Thus far, the results demonstrate the excellent tradeoff of FCPA strategy for the analyzed scenarios.

### 6.6. Sixth Scenario

**Figure 14:**
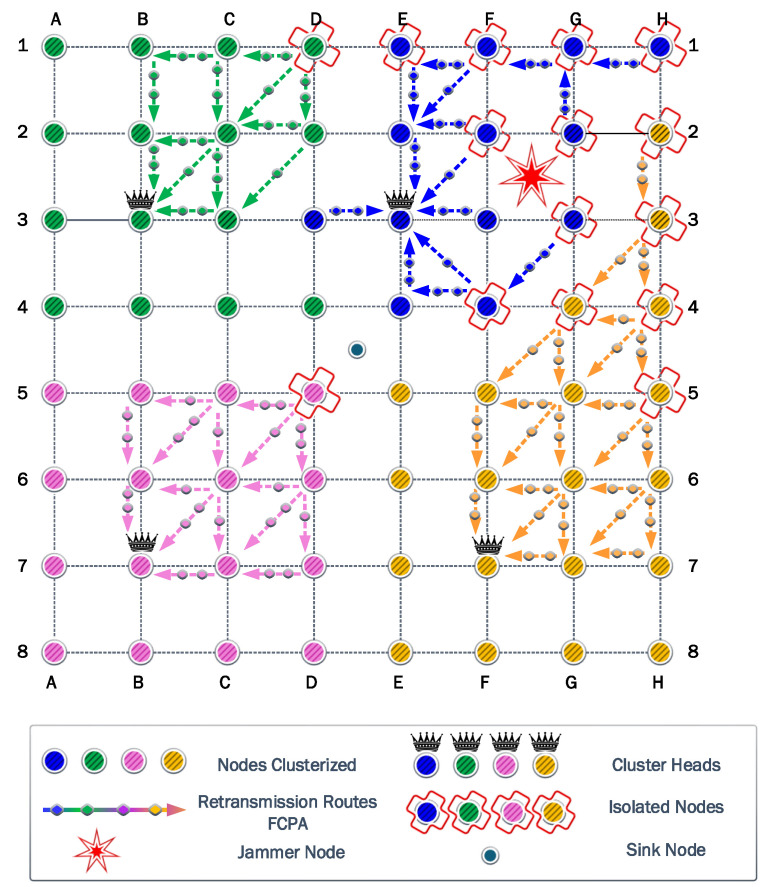
Sixth scenario: where the jammer is in the quadrant EG23, several nodes from the blue cluster are now associated with the CH of the yellow cluster. Additionally, one node from the green cluster decides to associate with the CH of the blue cluster.

In this scenario shown in Figure 14, the jammer is located in quadrant EG23, the CH of each cluster varies notably from the previous scenarios. The most affected cluster, the blue, changes its CH selection to E3. This change permits that node in position D3, which was previously from the green cluster, to now associate with the blue cluster. Additionally, several nodes in column H change their association to the yellow cluster.

The number of isolated nodes is 23.44% of total nodes in the network. As a consequence of the high impact of the jammer, the relay nodes will have a higher load to provide communication. On the other hand, the algorithm calculates that, with a transmission power of 23.45 dBm, the isolated nodes can communicate with the CH.

The information transmitted for FP and PA is only 199,171 kb, due to the high transmit power. The residual energy is 30.66 J for FP and 559.28 J for PA. The cooperation strategies use several relay nodes to connect all the nodes to the CH. This is explained by the relative distance between the jammer and the isolated nodes in column G. Note that as the associations that a relay can provide are reduced, the load per relay increases. The information transmitted for cooperation strategies is 94,937 kb for CP and 159,142 kb for PAC. The residual energy for these strategies is 559.28 J for CP and 566.80 J for PAC, with the worst tradeoff for the analyzed scenarios. Finally, the information transmitted for FCPA is 914,288 kb with a residual energy of 424.34 J. The main difference between the PAC cooperation strategy is the balanced use of the relays. As a consequence, the CH nodes exhaust their batteries first. However, in this scenario, the number of hops increases significantly for isolated nodes in column H.

### 6.7. Seventh Scenario

**Figure 15:**
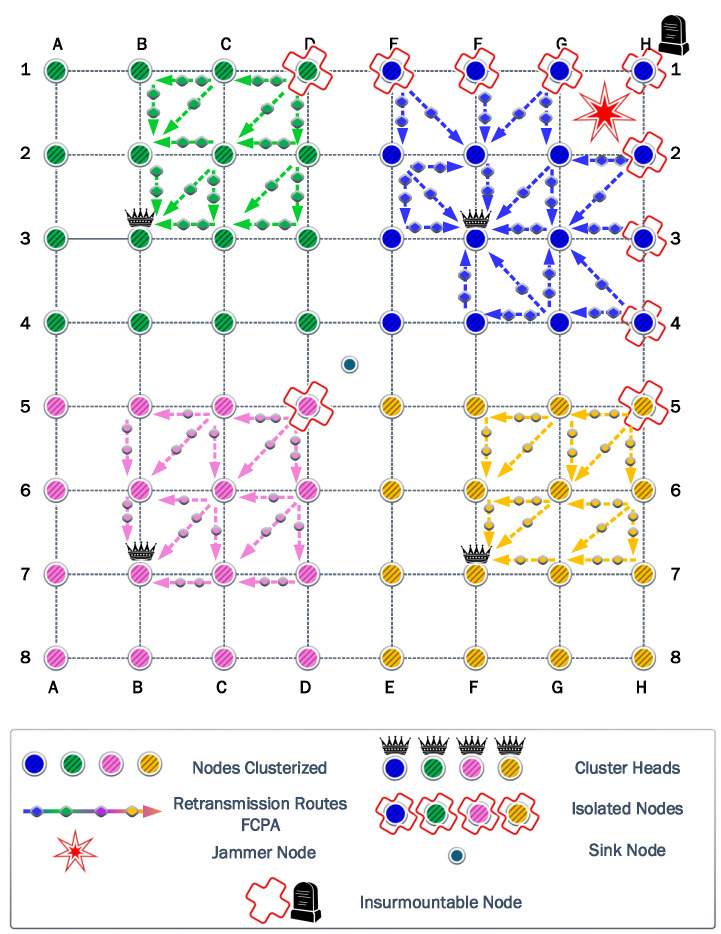
Seventh scenario: where, exceptionally, there exists a node that cannot communicate with any neighbor node.

Finally, the most damaging effects of the jammer happen when it is located in the quadrant GH12. For this scenario, the CH for each cluster is distributed in an asymmetrical form. However, the unique isolated nodes from the blue, green, and red clusters are in a perfect diagonal relative to their corresponding CH. However, only the blue cluster has 50% of isolated nodes. In Figure 15, a unique event occurs, the node H1 cannot communicate with any neighbor, using the maximum transmit power of 14 dBm. This node requires a transmit power of 18.28 dBm to reach the closest neighbor and 19.45 dBm to reach the CH.

For the cooperation strategies, the information transmitted is 245,868 kb for CP and 328,092 kb for PAC. Then, the residual energy is 484.50 J for CP and 517.78 J for PAC. Here, the relay nodes in positions E2 and G4 have the highest number of associations and exhaust their batteries first. Finally, the information transmitted for FCPA is 914,288 kb and the residual energy is 409.66 J. As for the cooperation strategies, the FCPA strategy cannot provide cooperation to node H1 owing to the maximum transmission power being limited by the transceiver hardware.

## 7. Main Findings

From the attacker’s point of view, the positions located near the contour of the network have certain benefits. In these positions, the attacker does not need to gain physical access to the property to perform the attack or deploy the jammer node. Therefore, scenarios four and seven extrapolate this condition and its impacts. Additionally, on average, the results show that the sixth scenario is the most detrimental to the WSN, with only 313,341 kb of information transmitted. On the other hand, the first scenario is the one with less residual energy at 56.66% , but the information transmitted is 629,810 kb. Considering the average results of the strategy on the analyzed scenarios, the power control domain surpasses the cooperation domain. The information transmitted for FP and PA is 571,963 kb versus the 137,976 kb and 215,354 kb by CP and PAC, respectively. Nevertheless, the residual energy of FP is very low, with a final value of 2.06% . Consequently, the self-healing strategy selection must consider the trade-off between WSN performance metrics.

FCPA stands out among self-healing strategies with 967,961 kb of information transmitted and 63% of residual energy on average. Whenever FCPA was used, the first node to exhaust its battery was a cluster head, demonstrating the high energy efficiency of this strategy. The integration of multiple techniques that increase the efficient use of resources is essential in IoT networks with devices with limited resources. On the other hand, UAVs can assist self-healing strategies by data collection from small clusters.

**Figure 16:**
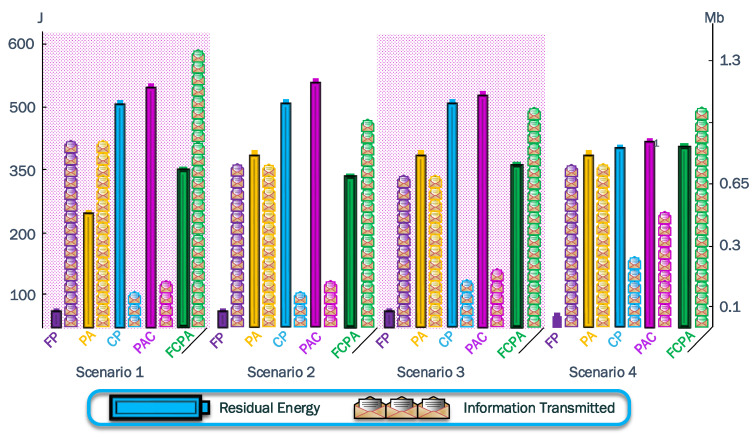
Bar plot for residual energy in J and information transmitted in kb for scenarios with the jammer moving along the *x*-axis. The closest difference between the bars from the same strategy shows a good tradeoff between the analyzed metrics. However, the information transmitted is the priority owing to the objectives of WSN.

**Figure 17:**
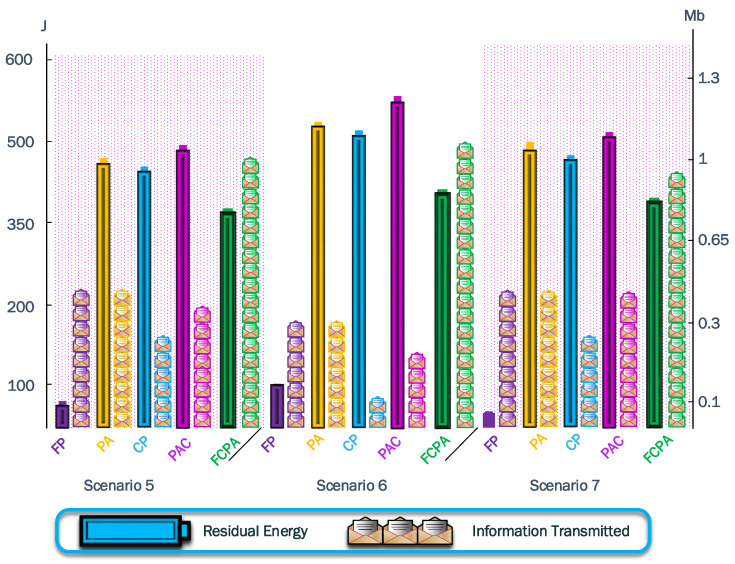
Bar plot for residual energy in J and information transmitted in kb for scenarios with the jammer moving diagonally across the cluster. The tradeoff is worse than the previous scenarios.

To summarize the results acquired from both metrics, we tabulate the data in Table 3 and show results in Figure 16 and Figure 17.

For all the scenarios, the FCPA strategy exhausts the batteries of the CH nodes. Therefore, it reaches the maximum information transmitted, while the residual energy is always over 50% for all scenarios.

Figure 16 shows the results from the jammer in the positions moving horizontally, while Figure 17 shows the results from the jammer in the positions moving diagonally. The algorithms FP, PA, CP, and PAC have a poor tradeoff since they cannot distribute the load evenly, so the best performance was always achieved by FCPA. In Figure 17, the tradeoff is worse than in Figure 16. Consequently, the jammer moving into a formed cluster notably impacts the integrity of the cluster and its communications.

## 8. Conclusions

In this study, we proposed a novel strategy of clustering and self-healing for WSNs under jamming attacks, called FCPA. On average, cooperation strategies with power control transmit two times more information than those with fixed power. However, power control strategies without cooperation exhaust the energy rapidly. But, despite the benefit of integrating both techniques, FCPA outperforms the other self-healing and clustering strategies in terms of information transmitted and residual energy, thanks to the load balancing that it implements.

Integrating cooperation and power control, named the PAC strategy, reduces the disadvantage of each approach. However, PAC does not balance the cooperation routes. Therefore, the network relay nodes spend their energy quickly, but FCPA effectively addresses this problem. The FCPA strategy surpasses the information transmitted by approximately 54.09% on average from the power control strategies in all scenarios. All the experiments confirmed that FCPA performs exceptionally in all the analyzed jamming scenarios.

In future work, we will evaluate different jamming strategies and propose strategies adaptable to them. We also require studying strategies that allow us to face the presence of intelligent and mobile jammers. Additionally, we intend to address artificial intelligence techniques to predict the effects of non-constant jamming and generate alternative paths.

## Figures and Tables

**Table 1:** Comparison with related work.

Reference	Power Allocation	Cooperation	Self-Healing	UAV	Jamming Scenarios
LEACH [8]	✓	-	-	-	-
SecLEACH [14]	✓	-	✓	-	-
Armor-LEACH [15]	✓	-	✓	-	-
MS-LEACH [16]	✓	-	✓	-	-
SEC [17]	✓	-	✓	-	-
Enhanced SLEACH [19]	✓	-	✓	-	-
Maheshwari [22]	-	✓	✓	-	-
Behera [23]	-	-	✓	-	-
Alazab [25]	-	✓	-	-	-
Sundararaj [31]	-	-	✓	-	-
Murugaveni [33]	-	✓	✓	-	✓
Bordon [34]	-	✓	-	-	-
Yabcznski [35]	✓	✓	-	-	-
Fascista [36]	-	-	-	✓	-
Just [37]	-	-	-	✓	-
Nazib [38]	-	-	-	✓	-
Almasoud [39]	-	-	✓	✓	✓
Li [40]	✓	-	-	✓	-
Our Proposal	✓	✓	✓	✓	✓

**Table 2:** System parameters used for the simulations.

Parameter	Value	References
Carrier Frequency	2400 MHz	[52]
Transmitter Height ( $h_{t}$ )	5 m	[53]
Receiver Height ( $h_{r}$ )	5 m	[53]
Intra Nodes Distance (*D*)	5 m	[15,53,54]
Path Loss Exponent ( α )	2	[44]
Noise Power Spectral Density ( $n_{0}$ )	−174 dBm/Hz	[34,35]
Message Length (*L*)	50 bits	[15,34,35]
Transmission Rate	200 kbps	[34,35]
Battery Charge	10 J	[34,35]
Power Amplifier Efficiency ( η )	0.35	[34,35]
Circuit Power Consumption	10 dBm	[35]
Maximum Transmit Power ( $P_{max}$ )	14 dBm	[5]

**Table 3:** Results of information transmitted and residual energy in percentage for all the scenarios analyzed in this work.

Scenario	Metric	FP	PA	CP	PAC	FCPA
1	Information Transmitted (kb)	804,528	804,528	118,192	139,158	1,280,000
	Residual Energy (%)	0.36	66.57	80.18	85.88	50.29
2	Information Transmitted (kb)	687,024	687,024	118,192	120,720	924,288
	Residual Energy (%)	1.22	70.81	85.23	89.45	62.03
3	Information Transmitted (kb)	602,422	602,422	118,192	192,278	914,288
	Residual Energy (%)	1.84	73.46	83.36	83.90	64.92
4	Information Transmitted (kb)	687,024	687,024	245,868	387,763	914,288
	Residual Energy (%)	1.22	72.27	72.77	75.08	72.27
5	Information Transmitted (kb)	534,835	534,835	245,868	300,262	914,288
	Residual Energy (%)	2.33	76.20	72.98	79.52	58.17
6	Information Transmitted (kb)	199,171	199,171	94,937	159,142	914,288
	Residual Energy (%)	4.79	87.39	88.56	88.90	66.30
7	Information Transmitted (kb)	488,739	488,739	245,868	328,092	914,288
	Residual Energy (%)	2.67	80.07	75.70	80.90	64.00

## Abbreviations

The following abbreviations are used in this manuscript:

BER	Bit Error Rate
CH	Cluster Head
CP	Cooperation Strategy
CSMA/CA	Carrier-sense multiple access with Collision Avoidance
DSSS	Direct-Sequence Spread Spectrum
DoS	Denial of Service
EECS	Energy-efficient Distributed Clustering
FCPA	Fairness Cooperation with Power Allocation Strategy
FHSS	Frequency-Hopping Spread Spectrum
FP	Fixed Power Strategy
HEED	Hybrid Energy Efficient Distributed
IoT	Internet of Things
IPN	Interference Plus Noise
LOS	Line of Sight
LEACH	Low-energy Adaptive Clustering Hierarchy
MAC	Medium Access Control
MRS	Minimum Receiver Sensitivity

NLOS	Non-line-of-sight
O-QPSK	Offest-Quadrature Phase Shift Keying
PA	Power Allocation Strategy
PAC	Power Allocation with Cooperation Strategy
PEGASIS	Power-Efficient Gathering in Sensor Information Systems
PDR	Packet Data Rate
PHY	Physical
QoS	Quality of Service
RSS	Received Signal Strength
RSSI	Received Signal Strength Indicator
SINR	Signal Interference-plus-Noise Ratio
SNR	Signal to Noise Ratio
TCCA	Time-Controlled Clustering Algorithm
TDMA	Time Division Multiple Access
TEEN	Threshold sensitive Energy Efficient sensor Network
UAV	Unmanned Aerial Vehicle
UWB	Ultrawide Band Technology
WSN	Wireless Sensor Network
