# Correction: López-Vilos et al. Clustering-Based Energy-Efficient Self-Healing Strategy for WSNs under Jamming Attacks. *Sensors* 2023, *23*, 6894

**DOI:** 10.3390/s24020628

**Published:** 2024-01-19

**Authors:** Nicolás López-Vilos, Claudio Valencia-Cordero, Richard Demo Souza, Samuel Montejo-Sánchez

**Affiliations:** 1 Synopsys Lisboa, 2740-267 Porto Salvo, Portugal; nicolasl@synopsys.com; 2 Department of Electrical Engineering, Universidad de Santiago de Chile, Santiago 9170124, Chile; claudio.valenciac@usach.cl; 3 Department of Electrical and Electronics Engineering, Federal University of Santa Catarina, Florianópolis 88040900, SC, Brazil; richard.demo@ufsc.br; 4 Programa Institucional de Fomento a la I+D+i, Universidad Tecnológica Metropolitana, Santiago 8940577, Chile

## Additional Affiliation

In the published publication [1], there was an error regarding the affiliation for Nicolás López-Vilos. The following revision is made in our published paper [1]:

^1^  Synopsys Lisboa, 2740-267 Porto Salvo, Portugal

The authors state that the scientific conclusions are unaffected. This correction was approved by the Academic Editor. The original publication has also been updated.
